# Differences in femur geometry and bone markers in atypical femur fractures and the general population

**DOI:** 10.1038/s41598-021-03603-2

**Published:** 2021-12-17

**Authors:** Ik Jae Jung, Ji Wan Kim

**Affiliations:** 1 University of Ulsan College of Medicine, Seoul, Republic of Korea; 2 Department of Orthopedic Surgery, Asan Medical Center, University of Ulsan College of Medicine, 88 Olympic-ro 43-gil, Songpa-gu, Seoul, 05505 Republic of Korea

**Keywords:** Endocrine system and metabolic diseases, Trauma, Geriatrics

## Abstract

This study aimed to identify differences in femur geometry between patients with subtrochanteric/shaft atypical femur fractures (AFFs) and the general population, and to evaluate the biomechanical factors related to femoral bowing in AFFs. We retrospectively reviewed 46 patients. Data on age, and history and duration of bisphosphonate use were evaluated. Femur computed tomography images were reconstructed into a 3D model, which was analyzed with a geometry analysis program to obtain the femur length, femur width and length, and femoral bowing. Patients were divided into two groups according to fracture location: the subtrochanteric and shaft AFF groups. We compared all parameters between groups, and also between each group and a general population of 300 women ≥ 60 years. Thirty-five patients had a history of bisphosphonate use (average duration, 6.1 years; range, 0.8–20 years). There was no statistical difference in bone turnover markers between the two groups. The shaft AFF group had a lower radius of curvature (ROC) (P = 0.001), lower bone mineral density (BMD, *T* score) (P = 0.020), and lower calcium (P = 0.016). However, other parameters and rate of bisphosphonate use were not significantly different. There were no significant differences in the parameters of the subtrochanter AFF group and the general population, but the shaft AFF group demonstrated a wider femur width (P < 0.001), longer anteroposterior length (P = 0.001), and lower ROC (P < 0.001) than the general population. Femoral bowing and width increased in shaft AFFs, but similar to subtrochanter AFFs compared to the general population. Our results highlight the biomechanical factors of femur geometry in AFFs.

## Introduction

The American Society for Bone and Mineral Research (ASBMR) defines atypical femur fracture (AFF) as a complete or incomplete fracture with major features, such as an atraumatic or minimal-trauma fracture, a transverse or short oblique orientation, a medial spike when the fracture is complete, non-comminuted or minimally comminuted, and localized periosteal or endosteal thickening of the lateral cortex (Table 1)^1^. Since Odvina et al.^2^ reported that AFF is associated with severe suppression of bone turnover after long-term use of bisphosphonate (BP) in 2005, BP has been suggested to be the main cause of AFF^3^. However, the second report of ASBMR showed that Asian ethnicity is a potential contributor to AFF risk^1^.

**Table 1:** ASBMR Task Force 2013 revised case definition of AFFs.

To satisfy the case definition of AFF, the fracture must be located along the femoral diaphysis from just distal to the lesser trochanter to just proximal to the supracondylar flare
In addition, at least four of five major features must be present. None of the minor features is required but have sometimes been associated with these fractures
**Major features**
The fracture is associated with minimal or no trauma, as in a fall from a standing height or less
The fracture line originates at the lateral cortex and is substantially transverse in its orientation, although it may become oblique as it progresses medially across the femur
Complete fractures extend through both cortices and may be associated with a medial spike; incomplete fractures involve only the lateral cortex
The fracture is noncomminuted or minimally comminuted
Localized periosteal or endosteal thickening of the lateral cortex is present at the fracture site (“beaking” or “flaring”)
**Minor features**
Generalized increase in cortical thickness of the femoral diaphysis
Unilateral or bilateral prodromal symptoms such as dull or aching pain in the groin or thigh
Bilateral incomplete or complete femoral diaphysis fractures
Delayed fracture healing

*ASBMR* American Society for Bone and Mineral Research, *AFF* atypical femur fracture.

Moreover, the role of low limb geometry has emerged as a risk factor for AFF^4^. Abnormal lower-limb geometry is thought to elevate stress within the lateral cortex of the femoral shaft and cause AFF by increasing mechanical fatigue, and numerous studies in East Asia support this explanation^5–8^. However, in a comparison of different ethnic groups in America, Asian populations showed an increased risk of AFF relative to other races^9–12^. Asian women have a more curved femur than Caucasians, while Africans tend to have the straightest femurs^13–15^, which would explain the high prevalence of AFF in Asians. It has also been reported that diaphyseal AFF is more common than subtrochanter AFF in Korean patients with AFF^6,16^.

However, there is a lack of evidence for a difference in femoral bowing between the general population and patients with AFF. We hypothesized that femoral bowing of the diaphyseal shaft in AFF is different from that in the general population. Therefore, we aimed to identify differences in the femur geometry, including femoral bowing, between patients with subtrochanteric/shaft AFF and the general population. We also sought to compare differences in bone turnover markers between patients with subtrochanter and shaft AFF.

## Materials and methods

### Study subjects

This study was approved by the Institutional Review Board of ASAN MEDICAL CENTER (protocol no. 2019-1568; November, 2019). The institutional committee waived the need for informed consent. The inclusion criteria were as follows: (1) presence of AFF, as defined by the ASBMR in 2014^1^, and (2) patients with full-scan computed tomography (CT) images of femurs, including an intact femur. Patients with peri-implant fractures (n = 2), periprosthetic fractures (n = 1), or bone metastasis (n = 0) were excluded. Initially, among 92 AFF patients screened from a single center between 2015 and 2020, 46 patients with AFF were enrolled. All patients were women with > 60 years of age, with an average age of 75.1 years (range, 61–90 years). A subtrochanteric fracture was defined as a fracture extending up to 5 cm below the lesser trochanter. A shaft fracture was defined as a fracture extending from the subtrochanteric region to the supracondylar metaphyseal flare. Data, including age, body mass index (BMI), bone mineral density (BMD), and history and duration of BP use, were evaluated. BMD was measured using the Prodigy dual-energy X-ray absorptiometry system (Advance™, GE-Lunar, Madison, WI, USA).

### Analysis of femur geometry using a 3D model

**Figure 1:**
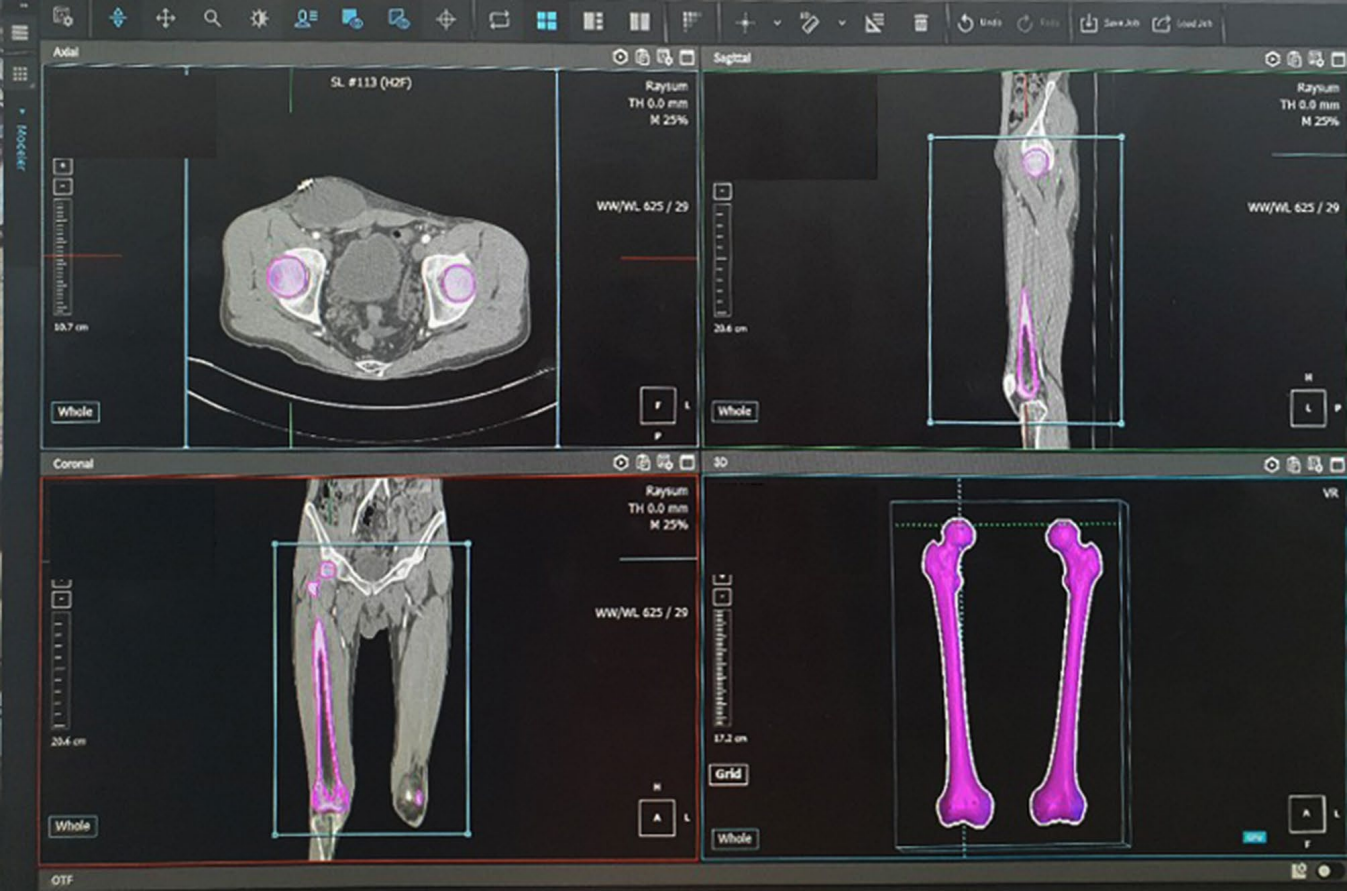
Masking, segmentation, and solid modeling procedure.

**Figure 2:**
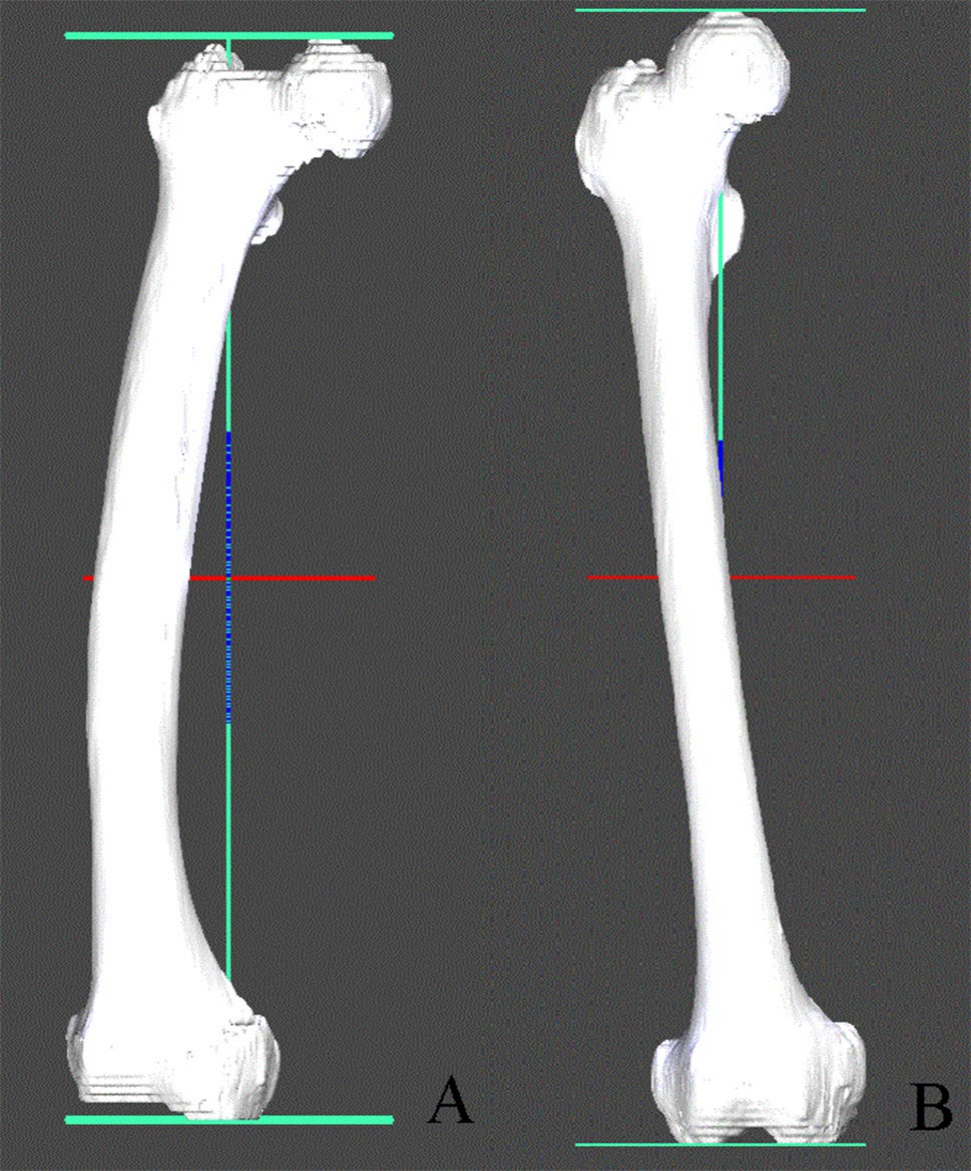
Femur length of the 3D model. (**A**) Contralateral femur of the patient with femur AFF. (**B**) Contralateral femur of the patient with subtrochanteric AFF.

**Figure 3:**
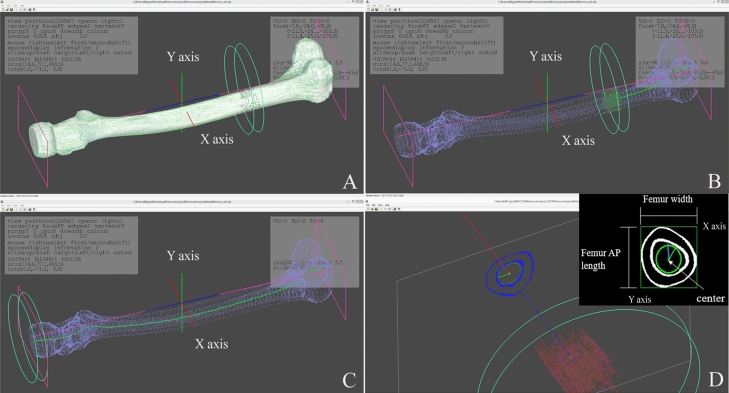
Depiction of the analysis. (**A**) Determination of solid model. (**B**) Identification of the center of the cross-sectional area with a rendered and transparent model. (**C**) Termination of the identification of the center at all level of the femur. (**D**). Measurement the medullary canal, femur AP.

The participants’ femur CT images were imported into a 3D modeling software (AVIEW Modeler; Coreline Soft, Seoul, South Korea) to produce 3D samplings of anatomical elements of the femur (Fig. 1). With the use of reconstruction and parametrization from these datasets, the structured data were segmented in obj format to form a 3D model representing both the bone surface and the corticocancellous interface (Fig. 2). The geometry analysis program using skeletonization was developed and conducted to obtain compact representation of the femur^17^. The following femur geometric parameters were calculated: (1) femur length from the upper pole of the femoral head to the bicondylar baseline (Fig. 3); (2) femur shaft length from the tip of the greater trochanter to the bicondylar line; (3) the narrowest diameter of the medullary canal; (4) anteroposterior length and lateral width of the entire femur; and (5) radius of curvature (ROC) of the femur, including the degree of bowing. Parameters 3, 4, and 5 were calculated from the level of the lesser trochanter to the level of the supracondylar area.

### Measurement of bone turnover markers (BTMs)

The serum concentration of 25OH-Vit.D3 was measured by radioimmunoassay using DIAsource 25OH-Vit.D3-Ria-CT Kit (DIAsource ImmunoAssays S.A.; Lauvain-la-Neuve, Belgium). The intra- and inter-assay coefficients of variation (CVs) were 7.2–8.7% and 7.2–7.3%, respectively. Bone turnover markers (BTMs), including serum osteocalcin, parathyroid hormone (PTH), C-telopeptide (CTX), Ca, P, alkaline phosphatase (ALP), and albumin, were reviewed. The serum calcium level was measured using the o-Cresolphthalein-complex one with a Toshiba 200FR autoanalyzer (Toshiba Medical Systems, Tokyo, Japan), and serum ALP was also measured. The serum intact PTH level was measured using an ELSA-PTH immunoradiometric assay (Cisbio Bioassays., Codolet. France), with a lower limit of detection of 0.7 pg/ml. The intra- and inter-assay CVs were 4.2–7.5% and 3.4–6.8%, respectively. The serum C-terminal telopeptide of type I collagen (CTX; normal range, 0.556 ± 0.226 ng/mL) concentration was measured using an electrochemiluminescence immunoassay on a Cobas 8000 e602 analyzer (Roche Diagnostics, Mannheim, Germany) with intra- and inter-assay CVs of 2.0–5.5% and 2.7–7.6%, respectively. The serum osteocalcin (OC; normal range, 15–46 ng/mL) concentration was also measured using an electrochemiluminescence immunoassay on a Cobas 8000 e602 analyzer (Roche Diagnostics) with intra- and inter-assay CVs of 0.7–0.8% and 1.5–1.6%, respectively.

### Statistics analysis

Patients were divided into two groups according to fracture location: group 1, with subtrochanteric AFFs, and group 2, with shaft AFFs. All parameters were compared between the two groups, and femur geometry parameters were compared between each group and a general population of 300 women ≥ 60 years, which was published in our previous study^17^. Descriptive statistics were used to determine the group means and standard deviations for numerical data. Each parameter was compared using a *t*-test. To identify risk factors of AFF, multivariate regression analysis was performed. Statistical significance was defined as a *P-*value < 0.05. Statistical analysis was performed using SPSS version 23.0 (IBM Corp., Armonk, NY, USA).

### Ethical approval

All procedures involving human participants were performed in accordance with the ethical standards of the institutional committee.

### Informed consent

The institutional committee waived the need for informed consent.

## Results

There were 20 patients with subtrochanteric AFF and 26 with shaft AFFs. The average height and weight of the patients were 150.9 cm (range, 135.0–168.4 cm) and 53.7 kg (range, 36.6–70.5 kg). The average BMI was 23.8 kg/m^2^ (range, 16.9–35.7 kg/m^2^). Thirty-five patients (76.1%) had a history of BP use, with an average duration of 6.1 years (range, 0.8–20 years). The rate of the history of BP use was not significantly different between the two groups. BMD was evaluated in 42 patients (91.3%); the mean *T* score of BMD was − 3.1 (range, − 1.5 to − 7.0), and 32 patients had osteoporosis (*T* score ≤ − 2.5) and 10 had osteopenia (− 2.5 < *T* score < − 1.0). The mean serum 25(OH) Vit. D level was 33.8 ng/ml (range, 7.6–66.7 ng/ml), and the rate of below 20 ng/ml was 23.3%. The mean serum CTX level was 0.26 ng/ml (range, 0.03–0.67), and the rate of lower level CTX (< 0.104 ng/ml) was 23.7%. BTM showed no statistical difference between the two groups (Table 2).

**Table 2:** Comparison between subtrochanter fracture and shaft fracture.

	Subtrochanter, n = 20	Shaft, n = 26	Difference	*P* value
Age, yr	74.0 ± 7.4	76.0 ± 6.9	− 2.1 ± 2.1	0.328
Femur length, mm	395.6 ± 21.3	405.5 ± 25.9	− 9.9 ± 7.1	0.173
Femur shaft length, mm	356.0 ± 20.5	366.2 ± 21.2	− 10.3 ± 6.2	0.106
Femur width, mm	27.9 ± 2.2	29.0 ± 1.8	− 1.12 ± 0.6	0.065
Femur AP length, mm	27.0 ± 1.5	28.0 ± 2.4	− 1.0 ± 0.6	0.108
Narrowest medullary diameter, mm	8.8 ± 1.7	9.1 ± 2.0	− 0.3 ± 0.6	0.597
ROC, mm	783.8 ± 108.9	642.3 ± 171.8	141.5 ± 41.6	0.001
Bisphosphonate use, %	80.0 (16/20)	73.1 (19/26)	–	0.732
Bisphosphonate use duration, years	6.4 ± 6.1	5.9 ± 5.1	0.4 ± 1.9	0.820
BMD (*T* score)	− 2.7 ± 1.3	− 3.5 ± 0.8	0.8 ± 0.3	0.020
Ca, mg/dl	9.3 ± 0.8	8.7 ± 0.6	0.6 ± 0.2	0.016
P, mg/dl	3.5 ± 0.9	3.4 ± 0.8	0.1 ± 0.3	0.684
25(OH) Vit-D, ng/ml	36.7 ± 16.6	31.7 ± 14.0	4.9 ± 4.7	0.298
PTH, pg/ml	46.8 ± 47.9	47.4 ± 28.7	− 0.6 ± 11.7	0.961
Osteocalcin	8.4 ± 7.2	7.2 ± 6.4	1.2 ± 1.8	0.517
Osteocalcin < 15, %	94.4 (17/18)	87.5 (19/23)	–	0.363
CTX	0.23 ± 0.20	0.27 ± 0.15	− 0.04 ± 0.05	0.436
CTX < 0.104 (− 2SD), %	38.8 (7/18)	16.7 (4/24)	–	0.159

*AP* anteroposterior, *ROC* radius of curvature, *BMD* bone mineral density.

The average femur length was 401.2 ± 24.3 mm and the average femur shaft length was 361.8 ± 21.3 mm. The average femur width was 28.6 ± 2.1 mm and the average femur AP diameter was 27.6 ± 2.1 mm. The average narrowest diameter of the medullary canal was 9.0 ± 1.9 mm. The average ROC was 703.8 ± 162.6 mm.

In a comparison of the fracture location in AFF, the shaft AFF group had a lower ROC (642.3 mm vs 783.8 mm, P = 0.001), lower BMD (*T* score) (− 3.5 vs − 2.7, P = 0.020), and lower calcium (8.7 mg/dl vs 9.3 mg/dl, P = 0.016). However, the other parameters were not significantly different (Table 2).

Table 3 shows the comparison between the subtrochanter AFF group and the general elderly population. None of the parameters of the subtrochanter AFF group were significantly different compared to the general population. However, the shaft AFF group had wider femur width (29.0 mm vs 27.5 mm, P < 0.001), greater AP length (28.0 mm vs 26.7 mm, P = 0.001), and lower ROC (642.3 mm vs 820.5 mm, P < 0.001) than the general population (Table 4). Table 5 summarized the risk factors of AFF. Among the variables including age, BMI, and femur geometry factors, femur width, AP length, and ROC was significant factors in the shaft AFF, but there were no significance in the subtrochanter AFF.

**Table 3:** Comparison between subtrochanter fracture and female population (> 60 yr).

	Subtrochanter, n = 20	Population (F, > 60 yr), n = 300	Difference	*P* value
Age, yr	74.0 ± 7.4	74.3 ± 8.5	− 0.4 ± 1.9	0.845
Femur length, mm	395.6 ± 21.3	401.2 ± 20.2	− 5.6 ± 4.7	0.235
Femur shaft length, mm	356.0 ± 20.5	361.4 ± 19.2	− 5.4 ± 4.5	0.226
Femur width, mm	27.9 ± 2.2	27.5 ± 2.1	0.4 ± 0.5	0.352
Femur AP length, mm	27.0 ± 1.5	26.7 ± 1.8	0.3 ± 0.4	0.494
Narrowest medullary diameter, mm	8.8 ± 1.7	9.5 ± 1.7	− 0.7 ± 0.4	0.105
ROC, mm	783.8 ± 108.9	820.5 ± 154.2	− 36.7 ± 35.1	0.296

*AP* anteroposterior, *ROC* radius of curvature.

**Table 4:** Comparison between shaft fracture and female population (> 60 yr).

	Shaft, n = 26	Population (F, > 60 yr), n = 300	Difference	*P* value
Age, yr	76.0 ± 6.9	74.3 ± 8.5	1.7 ± 1.7	0.318
Femur length, mm	405.5 ± 25.9	401.2 ± 20.2	4.3 ± 4.2	0.308
Femur shaft length, mm	366.2 ± 21.2	361.4 ± 19.2	4.9 ± 4.0	0.222
Femur width, mm	29.0 ± 1.8	27.5 ± 2.1	1.6 ± 0.4	< 0.001
Femur AP length, mm	28.0 ± 2.4	26.7 ± 1.8	1.3 ± 0.4	0.001
Narrowest medullary diameter, mm	9.1 ± 2.0	9.5 ± 1.7	− 0.4 ± 0.4	0.322
ROC, mm	642.3 ± 171.8	820.5 ± 154.2	− 178.2 ± 31.8	< 0.001

*AP* anteroposterior, *ROC* radius of curvature.

**Table 5:** Risk factors of AFF according to the fracture location.

	Subtrochanter, n = 20			Shaft, n = 26		
	*P* value	Odds ratio	95% CI	*P* value	Odds ratio	95% CI
Age, yr	0.948	1.002	0.942–1.066	0.726	0.988	0.922–1.058
BMI	0.393	1.054	0.934–1.190	0.061	0.860	0.724–1.007
Femur shaft length	0.168	0.978	0.947–1.009	0.076	1.031	0.997–1.067
Femur width	0.218	0.218	0.913–1.490	0.002	1.568	1.177–2.090
Femur AP length	0.248	0.248	0.878–1.655	0.025	1.395	1.042–1.868
Narrowest medullary diameter	0.114	0.114	0.566–1.063	0.224	0.823	0.602–1.126
ROC	0.663	0.663	0.996–1.003	< 0.001	0.989	0.985–0.994

*BMI* body mass index, *AP* anteroposterior, *ROC* radius of curvature.

## Discussion

Our results demonstrate that the femurs of patients with shaft AFFs were more bowed than those with subtrochanter AFFs, which agrees with the findings of previous studies^5,8,18–22^. Mechanically, the lateral cortex of the femur needs to endure tensile stress because of bending. The bending stress becomes severe when the femur is more bowed. The CT-based finite element analysis showed a correlation between femoral bowing and maximum principal stress, which indicates tensile stress^5^. Moreover, patients with mid-shaft AFF, who have more bowed femurs, showed marked diffuse tensile stress in the anterolateral surface of the shaft. In these patients, CT-based finite element analysis showed maximum tensile stress adjacent to the level of the fracture site in the mid-shaft. Oh et al. presented that a significantly higher lateral bowing angle and anterior radius of curvature of the femur in shaft AFF was related with a higher maximum principal stress (weakest fracture resistance) in a prospective finite elementary analysis (FEA) study of 18 patients^23^. Haider et al. showed a comparable result using 2-level full factorial analysis of an FEA of 10 patients in 2018^24^. Many previous studies, and the current results, have identified an association between femoral curvature and fracture location; therefore, we can conclude that increased elevated femoral bowing is associated with a more distal fracture location.

To the best of our knowledge, this is the first study to compare femur geometry between patients with AFF and a general elderly population. The results showed that there were differences in the femur geometry of patients with shaft AFF, but not those with subtrochanter AFFs compared to the general population. Patients with shaft AFF had wider femurs because of osteoporotic changes caused by the aging process^25^. BMD also decreases with the aging process of bone, leading to a decline in cortical area and bone strength. This chemical change in bone composition subjects the lateral cortex of the femur to greater bending stress. The compensation to overcome this bending stress increases the width of the femur because the bending strength of the bone is proportional to its area moment of inertia^26,27^. Therefore, a wider shaft diameter would reflect a decrease in BMD, which increases the risk of insufficiency fractures, including AFF. Both femoral bowing and shaft diameter impact the magnitude and volume of the strain in the femoral shaft^24^. The more bowed and wider the femoral shaft is, the more it will be exposed to stronger tensile stress at mid-shaft biomechanically, and tensile stress by loading stress can cause the development of AFF^5^. Compared to the general population, the femur geometry of patients with shaft AFF is more vulnerable to repetitive significant tensile stress in the lateral cortex around the fracture site. Therefore, shaft AFF could occur as a result of a mechanical factor of different femoral geometry regardless of a state of BP-induced oversuppressed bone turnover.

A recent study of AFF with bone metabolism markers and histological analysis also indicated that shaft AFF is an insufficiency fracture (stress fracture). Biological activity tends not to be suppressed in mid-shaft stress fractures of the bowed femoral shaft, while subtrochanteric AFFs involve bone turnover suppression^28^. This previous study also revealed that the level of the bone resorption marker, serum tartrate-resistant acid phosphatase-5b (TRACP-5b), was significantly lower in the subtrochanteric AFF group than in the mid-shaft AFF group, while the levels of the bone formation markers, N-terminal propeptide of type 1 procollagen and bone alkaline phosphatase (BAP) were not statistically different between shaft (n = 18) and subtrochanter AFF (n = 19) groups. Two-thirds of the patients with AFF had a history of BP or denosumab use, but histological analysis revealed that shaft AFFs showed active bone remodeling. In our study, there was no significant difference in BP use, or bone resorption and formation markers, although we have no histological data. Therefore, considering that the use of BP and the resulting decrease in bone turnover markers do not necessarily indicate “over-suppression” of bone remodeling, further understanding of the “femur geometry of bowing” will shed new light on the etiology of AFFs.

A recent study by our group^17^ revealed that the femur in Asian (Korean) elderly women was more bowed than that in young women or elderly men. This study showed that the femur in shaft AFF was much more bowed than that of general population elderly women who have more bowed femurs than young women or elderly men. The result suggested that femurs with severe bowing are prone to insufficiency femur shaft fracture, a type shaft AFF. Additionally, there is some evidence to suggest that individuals with diaphyseal AFF tend to be older and weigh less than those with subtrochanteric AFF^4,6,21,29^. Therefore, the femoral geometric characteristics of Asian populations may explain why more shaft AFFs occur in Asians, even in BP‐naïve patients, although over-suppression of bone remodeling by BP use is still considered the main reason for AFFs.

This study has several limitations. First, we have no data to indicate the bone turnover state, such as BMD or duration of BP use, in the general population. Therefore, it is difficult to compare BP use or the bone remodeling state population. In future, a study about etiology of AFF related with correlation between the use of bisphosphonates and femoral geometry would be clarified. Second, the current data only included bone turnover markers, and not histological findings, which limited our ability to evaluate the biological activity of bone remodeling. In the near future, we intend to perform a comparison of the biological activity of bone remodeling and bone turnover markers between patients with AFF and the general populations. Finally, this study is retrospective nature and compared with a small cohort concerning the general population published in a previous study. Greater patient numbers could provide more information in validation, but rarity of AFF limits enrollment. Despite these limitations, this study is the first study to compare the femur geometry of patients with AFF to that of the general population. As a result of our findings, we suggest the possibility that shaft AFF is an insufficiency (stress) fracture.

## Conclusion

Many patients (76%) with AFF had history of the use of BP regardless of the fracture location. This geometry analysis demonstrated that femoral bowing and increased width is observed in shaft AFFs, but similar to subtrochanter AFFs compared to the general population. Our results highlight the biomechanical factors of femur geometry as an etiology of AFFs.

## Data Availability

We did not add supplement data and materials in submission but are available in request to the corresponding author if it is necessary for review.
